# Multiaxial Fatigue Life Assessment of Large Welded Flange Shafts: A Continuum Damage Mechanics Approach

**DOI:** 10.3390/ma18245528

**Published:** 2025-12-09

**Authors:** Zhiqiang Xu, Chaolong Yang, Feiting Shi, Wenzheng Liu, Na Xu, Zengliang Hu, Chuanqi Li, Ketong Liu, Peng Cao, Di Wang

**Affiliations:** 1 School of Chemical and Machinery, Liaodong University, Dandong 118001, China; 2 College of Architecture and Civil Engineering, Beijing University of Technology, Beijing 100124, China; 3 School of Civil Engineering, Yancheng Institute of Technology, Yancheng 224051, China; 4 Jiangsu Engineering and Research Center of Intelligent Disaster Prevention in Coastal Transportation Infrastructure, Yancheng 224051, China; 5 College of Architecture and Civil Engineering, Xi’an University of Science and Technology, Xi’an 710054, China

**Keywords:** large welded flange shaft, high-cycle fatigue, continuum damage mechanics, multiaxial fatigue, life prediction

## Abstract

This study develops a unified continuum damage mechanics (CDM) model for high-cycle fatigue life prediction of large manually arc-welded flange shafts manufactured from 45Mn steel (quenched and tempered) under combined bending–torsion loading. Fatigue tests revealed consistent crack initiation at the weld toe, with multiaxial loading reducing fatigue life by 35–42% compared to pure bending. The CDM parameters were calibrated against experimental data and implemented through an ABAQUS 2021 UMAT subroutine, achieving prediction errors below 5%—significantly outperforming conventional nominal and hotspot stress methods. For high-cycle fatigue conditions, a simplified CDM model neglecting plastic damage maintained engineering accuracy while improving computational efficiency by 3–5 times. The damage variable D = 0.9 was identified as a universal threshold for accelerated damage progression. These findings provide quantitative basis for multiaxial fatigue design and structural health monitoring of large welded components.

## 1. Introduction

Large welded flange shafts are key structural components in wind power equipment, marine propulsion systems, and heavy machinery transmission fields. Welded joints have been widely used in engineering structures due to their excellent mechanical properties and weldability [1]. However, under different loading conditions, the evolution mechanism of fatigue damage is relatively complex, leading to frequent fatigue cracks in the welding zone [2,3]. Their fatigue failure will not only cause catastrophic safety accidents but also result in substantial economic losses [4]. The susceptibility to fatigue in these regions is intrinsically linked to the physical metallurgy of the welding process [5,6].

The welding process introduces significant microstructural heterogeneity, residual stresses, and potential defects, which collectively dominate the fatigue behavior. Specifically, the rapid thermal cycles create a heat-affected zone (HAZ) with altered microstructures (e.g., coarsened or softened phases compared to the base metal) [5,7,8,9]. The weld toe and fusion line act as inherent stress concentrators, promoting crack initiation. Furthermore, the solidification of the weld pool can lead to defects, and the constrained cooling typically generates high tensile residual stresses in the weld region [10]. These factors—microstructural inhomogeneity, stress concentration, and residual stresses—synergistically deteriorate the fatigue resistance of the component. This deterioration is particularly severe under non-proportional multiaxial loading, where the interaction between evolving stress states and the initial welding imperfections is highly complex [11,12].

Current fatigue assessment standards, such as the nominal stress [13,14,15] and hot spot stress methods [16,17,18] recommended by IIW, Eurocode 3, and others, often fall short in addressing these coupled challenges. While methods like the notch stress [19,20,21] and strain energy density [22,23,24] offer improvements, they primarily rely on linear-elastic assumptions and are sensitive to modeling techniques (e.g., mesh dependency). Fracture mechanics approaches [25,26] require prior knowledge of crack size, limiting their application to crack-initiation life prediction. These traditional methods, largely based on uniaxial fatigue data, struggle to accurately characterize the combined effects of multiaxial stress states (especially non-proportional hardening), material nonlinearity, and the specific damage mechanisms originating from the weld microstructure. Consequently, life predictions can be overly conservative or dangerously non-conservative, as evidenced by field failures of welded shafts.

Continuum Damage Mechanics (CDM) provides a promising framework to overcome these limitations by directly modeling the damage evolution process from micro-crack initiation to macro-crack propagation [27,28]. Recent research has demonstrated the capability of CDM in predicting fatigue life for welded specimens, considering factors like porosity [29] and pitting damage [30,31]. However, a significant research gap remains in applying CDM to large-scale welded structures like flange shafts. The key challenge lies in effectively integrating the influences of welding-induced microstructural gradients and residual stresses into a practical CDM model that is validated under realistic multiaxial loading conditions (bending and torsion).

It is well-established that the welding process creates a heterogeneous mechanical property distribution across the joint [32,33]. The solidified weld metal, typically with a cast microstructure, and the heat-affected zone (HAZ), which may experience softening or hardening depending on the thermal cycle and base metal composition, exhibit yield strengths that can differ significantly from the base metal [34]. This heterogeneity, especially in the context of a base metal with a relatively low yield strength, is critical for high-cycle fatigue behavior. Fatigue cracks are likely to initiate in the region with the lowest fatigue resistance, which could be the softened HAZ or stress-concentrated weld toe, rather than in the base metal itself [35]. Traditional stress-based methods struggle to capture this complex failure mechanism shift. The continuum damage mechanics (CDM) approach proposed in this study, however, is particularly suited to address this challenge as it models damage evolution based on local stress–strain fields and material state, inherently accounting for the effects of mechanical heterogeneity on the fatigue damage process [36,37].

In summary, there is an obvious gap in current research: although CDM has theoretical advantages, most existing studies focus on small standard specimens at the material level, and CDM-based multiaxial fatigue models for large-scale welded engineering structures (e.g., flange shafts) are extremely scarce. More importantly, a complete engineering scheme is lacking—specifically, how to integrate model construction, parameter calibration, and test data of full-scale structures under multiaxial loads (e.g., bending and bending–torsion) to achieve accurate fatigue life prediction of such key components. This research gap is not only a current academic challenge but also an engineering bottleneck restricting the reliability design of high-end equipment. Therefore, this study aims to construct a CDM-based multiaxial fatigue life prediction model suitable for large welded flange shafts, realizing accurate prediction of their fatigue life under bending and bending–torsion combined loads. To achieve this goal, the main research contents include the following: (1) Conduct systematic fatigue tests on 45Mn steel welded flange shafts under bending and bending–torsion loads to obtain failure data and characterize their macro-failure characteristics. (2) Cite classical CDM theory to construct an evolution equation reflecting the damage mechanism of welded structures under multiaxial loads, and fit elastoplastic constitutive damage parameters based on test data. (3) Develop the corresponding UMAT user material subroutine, embed the established model into a finite element platform to realize numerical simulation of the damage evolution process, and verify model accuracy by comparing with test results. (4) Based on the validated model, systematically compare it with traditional methods, clarify the superiority of the CDM damage model in fatigue life prediction, and provide theoretical and technical references for the engineering design of large-scale welded structures.

## 2. Experiment Profiles

### 2.1. Test Materials and Specimen Preparation

**Figure 1:**
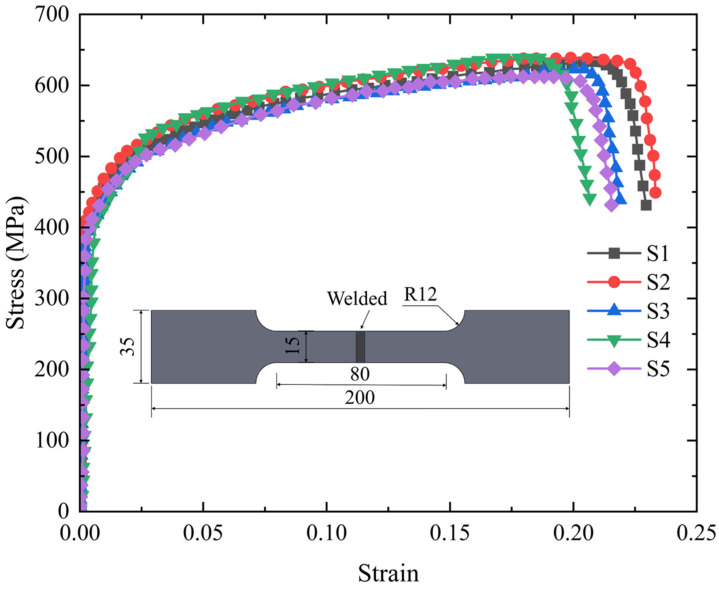
Geometric dimensions of the standard welding specimen and its stress–strain curve (unit: mm).

The base material for the flange shaft and flange plate specimens used in the fatigue tests of this study is 45Mn steel, sourced from the same batch as the material used in the author’s previous research [38], all purchased from Ansteel Co., Ltd. (Anshan, China). The forged flanges underwent normalization after forging to refine the grain structure and achieve a uniform ferrite-pearlite microstructure. The specified material (Certification No. B491201912) guaranteed consistency in composition and the resulting mechanical properties. The chemical composition of 45Mn steel is shown in Table 1, which meets the requirements of the GB/T 699-2015 standard [39]. According to the requirements of GB/T 228.1-2021 standard [40], similar steels to the welded flange shaft were selected, cut, and welded according to the standards into smooth dog-bone specimens S1–S5, with specific dimensions shown in Figure 1. Uniaxial tensile tests were conducted on specimens S1–S5 to determine their main mechanical properties such as elastic modulus, yield strength, and ultimate strength. Figure 1 presents the stress–strain curves obtained from the tests, and the three curves show no significant differences, indicating very minor differences in material properties among the specimens. Based on the stress–strain curve data, the main mechanical properties of the 45Mn steel welded flange shaft are shown in Table 2. These properties, including the yield strength, tensile strength, and elongation, are characteristic of the hot-rolled condition. Based on the steel’s carbon content and this thermo-mechanical processing history, the microstructure is expected to consist of a ferrite-pearlite mixture, which is the typical equilibrium microstructure for a medium-carbon steel under these conditions [41]. The welding electrodes used were E5015 low-hydrogen electrodes, which conform to the AWS A5.1/A5.1M-2012 standard [42], produced by Tianjin Jinqiao Welding Material Group Co., Ltd. (Tianjin, China), and their chemical composition and mechanical properties are also consistent with previous research [38], ensuring continuity of the welding process and stability of weld quality, and meeting the welding strength requirements for large flange shafts.

The carbon equivalent (CE) is a critical parameter for assessing the weldability of steel, as it indicates the susceptibility to cold cracking in the heat-affected zone (HAZ). Based on the provided chemical composition, the carbon equivalent was calculated using the International Institute of Welding (IIW) formula [43]:

(1) $${\mathrm{CE}}_{\left(\mathrm{IWW}\right)}=C+\frac{\mathrm{Mn}}{6}+\frac{\mathrm{Cr}+\mathrm{Mo}+V}{5}+\frac{\mathrm{Ni}+\mathrm{Cu}}{15}$$

The calculated CE value for the present 45Mn steel and E5015 electrodes are 0.581 and 0.553, respectively. Given that CE values exceeding approximately 0.40–0.45% significantly increase the risk of hydrogen-induced cold cracking, the high calculated values necessitate stringent preventive measures. Therefore, to ensure weld quality and mitigate cracking risks, a preheating temperature of 150–200 °C was rigorously applied before welding to slow the cooling rate and reduce the formation of hard, crack-sensitive microstructures (e.g., martensite) in the HAZ. Furthermore, a post-weld stress relief annealing was conducted to effectively mitigate the detrimental tensile residual stresses inherent in the welding process. The low contents of sulfur and phosphorus, as noted, contribute to good resistance to hot cracking and ensure high purity of the base metal, which is beneficial for overall toughness. The selection of the E5015 filler metal was based on a deliberate metallurgical strategy. Its low carbon content is essential for ensuring high toughness in the weld metal, while the elevated manganese content acts as a potent solid-solution strengthener to compensate for the strength loss and aids in desulfurization. This chemical design mitigates the cracking risk from the high-carbon base metal. Although the reviewer noted the S and P content, it is confirmed that these levels are within the acceptable limits specified for E5015 electrodes by the relevant AWS A5.1 standard. The primary design objective of this electrode is to achieve an optimal balance of strength and toughness, which is paramount for the fatigue performance of the welded structure. The low S and P contents of the base metal further contribute to good resistance to hot cracking and ensure high purity.

The specimen design is based on a large welded flange shaft engineering prototype, adhering to the three principles of “structural similarity, load adaptability, and easy installation.” On one hand, this ensures that the weld type, shaft–flange connection method, and the stress concentration area of the heat-affected zone (HAZ) of the weld are consistent with the actual engineering structure. On the other hand, it accommodates the loading limitations of the fatigue testing machine. The main goal is to use a standardized welding process and varied loading conditions to capture the effects of different loads on fatigue performance, thus providing multiple samples for the validation of the Continuum Damage Mechanics (CDM) model. All welded flange shaft specimens adopt the “separate shaft–flange forging plus manual arc welding connection” process: the flange shaft has a diameter of 60 mm and a length of 120 mm, with four rectangular smooth planes (h × b = 30 mm × 60 mm) machined at the end of the shaft to facilitate structural fixation and experimental loading. The flange plate has a diameter of 130 mm and a thickness of 20 mm, with eight bolt holes (each 13 mm in diameter, 15 mm from the edge of the flange) evenly machined along the circumference to allow for secure mounting to the fatigue testing machine’s base.

**Figure 2:**
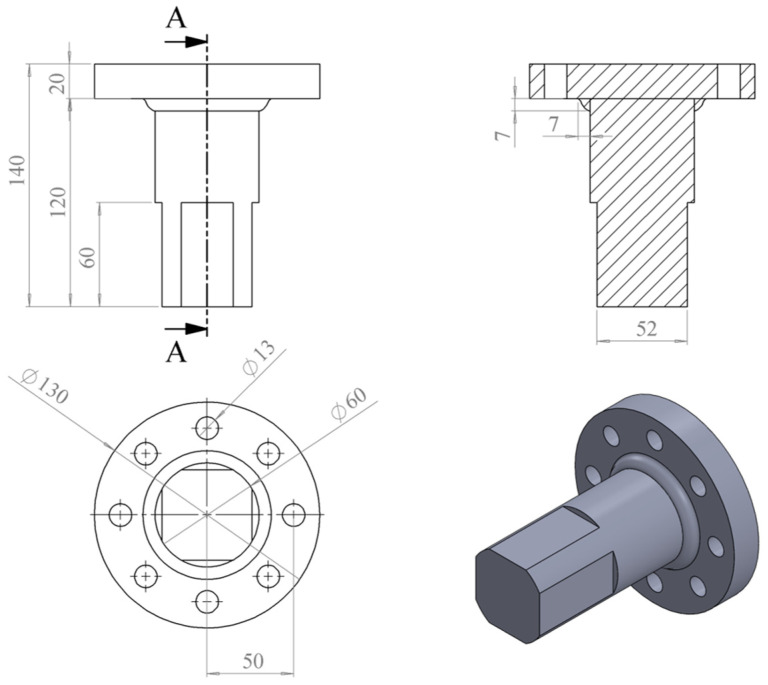
Dimensions of Welded Flange Shaft Specimen (unit: mm).

The flange plate and flange shaft were joined using a single-pass circumferential fillet weld fabricated by the Shielded Metal Arc Welding (SMAW) process. The target leg length of the fillet weld was 8 mm, and the weld toe was left in the as-welded condition, establishing the specific geometric configuration for the subsequent fatigue analysis. The welding parameters were as follows: a current of 170 A, a voltage of 24 V, and a travel speed of 60–90 mm/min. The specific protocol was as follows: prior to welding, the E5015 electrodes were baked at 350–400 °C for one hour and subsequently stored in a holding oven at 120–150 °C to minimize hydrogen introduction; a uniform preheat temperature of 150–200 °C was applied to the joint area before welding commencement; the interpass temperature was rigorously maintained within the range of 150–250 °C throughout the welding process; and a post-weld stress relief annealing was immediately conducted by heating the component to 590–610 °C, holding for two hours, followed by furnace cooling to below 300 °C before air cooling to room temperature. This stringent procedure was established based on the high carbon equivalent of the base metal and standard industry practices for welding high-strength steels. The welded specimen is shown in Figure 2.

### 2.2. Fatigue Test

In this experiment, a PWS-100 electro-hydraulic servo static-dynamic universal testing machine was used for fatigue loading. This testing machine, manufactured by Jinan Dongce Testing Machine Technology Co., Ltd. (Jinan, China), has a maximum static test force of ±100 kN, with an indication accuracy of 1–100% FS, and an error of ±0.5% at all points. The actuator’s maximum amplitude is ±75 mm, with an indication accuracy of ±0.5% FS. The loading frequency can be freely adjusted between 0.01 Hz and 20 Hz, fully meeting the loading requirements of this experiment. The testing machine is controlled by an electro-hydraulic servo structural fatigue test system. The fatigue tests in this study were conducted under force control with sinusoidal loading at a constant frequency of 4 Hz. This frequency was selected based on the following rationale: preliminary measurements verified that the resulting temperature rise on the specimen surface was negligible, thereby preventing any thermal influence on the material’s fatigue behavior in accordance with ASTM E466-2021 [44]. Furthermore, this frequency represents a practical balance for high-cycle fatigue testing, allowing all experiments to be completed within a reasonable timeframe while maintaining testing efficiency. The use of a uniform frequency across all specimens ensured full comparability of the fatigue life data by eliminating frequency as a potential variable.

**Figure 3:**
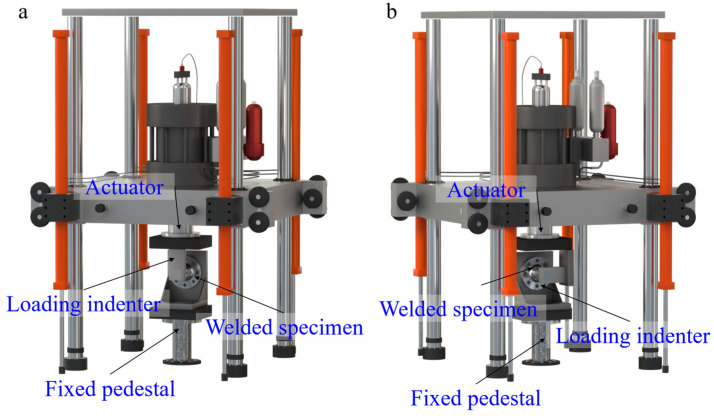
Schematic diagram of fatigue testing apparatus: (**a**) bending fatigue; (**b**) bending–torsion fatigue.

To ensure the reliability of test results, loading and environmental uncertainties were quantified based on the machine’s specifications: the applied load deviation (verified via 5 repeated calibrations) showed a coefficient of variation (CV) of 0.8% for bending loads and 1.2% for combined bending–torsion loads (both meeting GB/T 3075-2008 [45] requirements), while the actuator displacement error was within ±0.02 mm. All tests were performed in a constant temperature and humidity laboratory (23 ± 2 °C, 50 ± 5% RH), and pre-tests confirmed this environment’s influence on results was <1%. Additionally, 3 groups of identical specimens (n = 3, per group) were tested to verify repeatability: the CV of bending fatigue life was 2.3%, and that of combined bending–torsion fatigue life was 3.1% (both complying with fatigue test repeatability criteria), supported by uniform material properties, controlled welding parameters, and consistent specimen machining accuracy. The fatigue testing device and control system are shown in Figure 3.

The accuracy of the applied stress state, particularly under combined bending–torsion loading, is critically dependent on precise alignment to minimize spurious eccentricities. Therefore, the test fixture and specimen were carefully aligned using dial indicators to ensure coaxiality between the loading axis and the specimen. Prior to testing, alignment was verified by applying a small preload and confirming symmetric strain responses from strain gauges mounted at opposite positions. This procedure ensured that the induced stress state accurately represented the intended theoretical loading conditions.

In order to obtain the constitutive parameters for the CDM multi-axial fatigue damage model of large welded flange shafts and to verify the effectiveness of this model in predicting the fatigue life of welded flange shafts, multi-condition fatigue cyclic loading tests, such as bending and bending–torsion, were conducted on the specimens. Calibration of the material parameters in the relevant constitutive equations requires multi-condition fatigue test data under different cyclic stress levels. Therefore, a total of 24 large welded flange shaft specimens were divided into 8 groups, encompassing a variety of conditions such as different stress amplitudes and different fatigue loading modes, as shown in Table 3.

The fatigue test data of specimens under different working conditions were collected and organized. Using specialized fatigue testing equipment, the number of cycles each specimen underwent from the start of loading until fatigue failure was monitored and recorded in real time. This number of cycles represents the fatigue life. Table 3 shows the statistical results of the fatigue life for each specimen. Multiple parallel specimens were set under the same working conditions because fatigue tests themselves exhibit a certain degree of variability. By analyzing multiple parallel specimens statistically, the influence of this variability on the final results can be effectively reduced, making the test results more reliable and representative.

In order to further verify the validity of the fatigue test data, the mean value, standard deviation, and coefficient of variation in the logarithmic fatigue life were calculated using formulas, as shown in Table 4 below. According to the literature [46], when the sample size is 3, the limit value of $\left|\left(x_{i}-\bar{x}\right)/SE\right|$ is 1.53, in which case the mean values of $\left|\left(x_{i}-\bar{x}\right)/SE\right|$ for each stress level all meet the requirement of $\left|\left(x_{i}-\bar{x}\right)/SE\right|\leq 1.53$, and all data conform to the conditions.

(2) $$\bar{x}=\frac{1}{n}\sum x_{i},x_{i}=\mathrm{lg}N_{i}$$

(3) $$SE=\sqrt{\frac{1}{n-1}\left[\sum x_{i}^{2}-\frac{1}{n}{\left(\sum x_{i}\right)}^{2}\right]}$$

(4) $$\upsilon_{x_{i}}=\frac{SE}{\bar{x}}$$

where n is the sample size; $N_{i}$ is the fatigue life value corresponding to each samples; $\bar{x}$ is the mean value; SE is the standard deviation; $\upsilon_{x_{i}}$ is the coefficient of variation.

**Figure 4:**
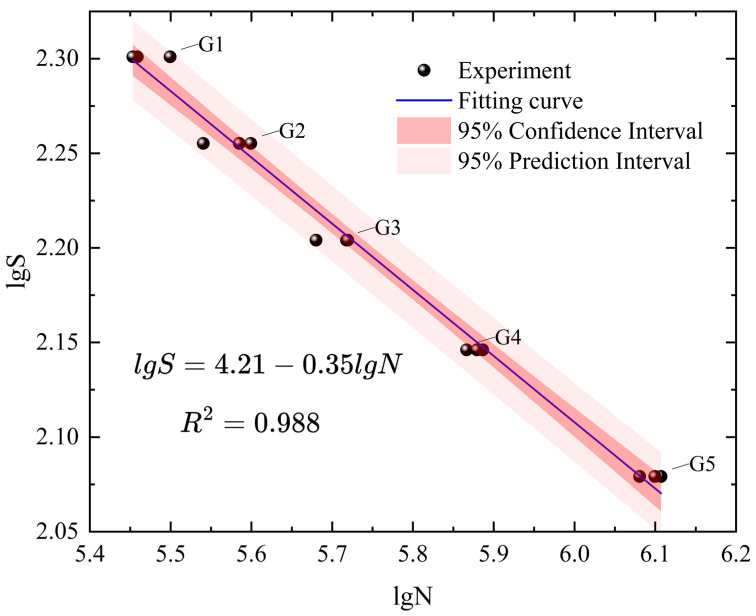
Logarithmic fitting and interval analysis diagram of the welded flange shaft bending fatigue S-N curve.

The fatigue test data for the five stress levels (G1-5) in Table 3 were plotted on a double-logarithmic graph and fitted accordingly. At the same time, the 95% confidence interval and 95% prediction interval of the fitted curve were calculated to quantify the uncertainty in the fatigue life assessment of the welded component, as shown in Figure 4. A linear equation was used to fit the test data on a log-log coordinate system, with a correlation coefficient of approximately 0.988 for the fitted function, indicating that using a linear function to fit the test data is appropriate. This agrees with the fundamental principle that the relationship between stress amplitude (S) and life (N) in the S-N curve for high-cycle fatigue of metallic materials is linear, further demonstrating the high accuracy and reliability of the testing method and experimental data used in this study. The figure shows that the width of the 95% confidence interval is relatively narrow within the full stress range of 120 MPa–200 MPa, indicating that the fitted S-N curve provides a high level of precision in estimating the overall average fatigue life of large welded flange shafts under bending fatigue conditions. In contrast, the width of the 95% prediction interval is significantly larger than that of the confidence interval. The reason for this is that the prediction interval reflects not only the uncertainty in estimating the population mean, but also the variability in fatigue life of individual welded flange shafts due to differences in residual stress distribution and the randomness of surface defects. Notably, at the maximum stress level of 200 MPa—which corresponds to the actual service conditions of the flange shaft—the range of the 95% prediction interval for fatigue life is from 1.04 × 10^6^ to 1.38 × 10^6^ cycles. This interval provides a direct and conservative engineering reference for determining the fatigue design life of the flange shaft, ensuring that there is a 95% probability the component will meet or exceed its design life requirements under actual bending service conditions, thereby avoiding the risk of equipment failure due to fatigue failure of welded structures. Moreover, the stability of the prediction interval’s width across the full stress range further confirms that, under bending fatigue loads of 120 MPa–200 MPa, the fatigue damage mechanism in large welded flange shafts primarily involves crack initiation and propagation at the welded joint, with no shift in damage mode being observed due to changes in stress level. Meanwhile, the relatively narrow 95% confidence interval indicates a high level of accuracy in estimating the overall “stress-life” relationship based on the sample data, providing a reliable data set to support fatigue performance evaluations of similar components in the future. The linear S-N relationship is fundamentally grounded in the fatigue mechanism of welded joints. The sharp weld toe acts as a potent stress concentrator, drastically reducing the crack initiation phase. Consequently, the total fatigue life is predominantly governed by crack propagation, a process well-described by a power law (Paris’ law) that manifests as a linear relationship on a log-log scale. This rationale is consistent with international standards (e.g., IIW recommendations [47], BS 7608 [48]) that prescribe linear S-N curves for welded components. The quantitative uncertainty intervals, summarized in Table 5, robustly supplement the graphical representation in Figure 4, providing a definitive basis for the model’s statistical precision and its utility in delivering conservative engineering design life estimates.

### 2.3. Macroscopic Failure Characteristics

**Figure 5:**
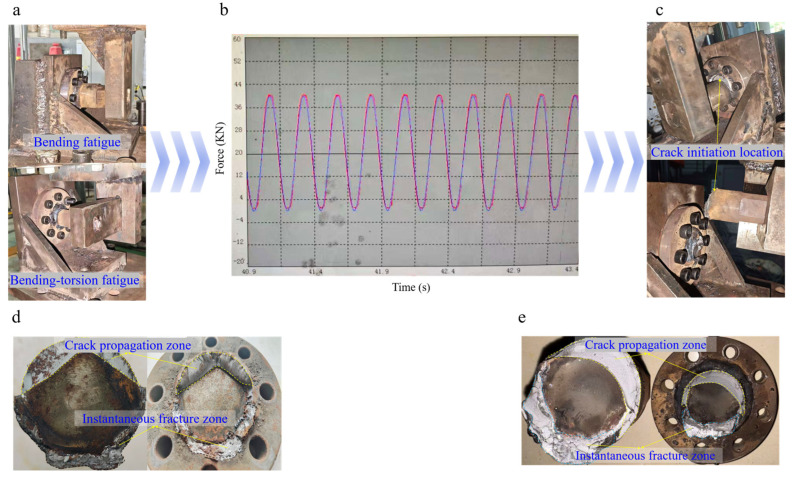
Experimental flow chart and macroscopic cross-section morphology of the specimen: (**a**) test mounting system; (**b**) test control system; (**c**) device diagram at the moment of specimen fracture; (**d**) fracture surface morphology of bending specimen; (**e**) fracture surface morphology of bending–torsion specimen.

The experimental flow charts and macroscopic fracture surface morphologies of the two loading conditions are shown in Figure 5. Observing the specimens after fatigue failure, obvious macroscopic failure characteristics can be found. Figure 5c presents the location of crack initiation and propagation in the flange shaft, from which it can be clearly observed that the initial cracks of the welded flange shaft initiate near the interface between the weld and the flange shaft—a high-stress region of the entire welded flange shaft. This observation aligns with the well-established understanding that the weld toe, due to the combined effect of geometric and microstructural discontinuities, is the most prevalent site for fatigue crack initiation in welded joints [49]. During the fatigue crack initiation stage, microcrack sources usually appear on the weld surface of the welded flange shaft, mostly concentrated in the stress concentration areas of the weld. Moreover, the inevitable initial defects in welding will act as crack initiation sources, significantly affecting the fatigue performance of the structure. With the continuation of cyclic loading, the cracks gradually propagate, leaving characteristic traces such as fatigue striations in the weld and adjacent areas of the flange shaft during the propagation process. Finally, when the cracks propagate to the critical size, the flange shaft undergoes sudden fracture. Observing Figure 5d,e, the fracture surface can generally be divided into the fatigue crack propagation zone and the instantaneous fracture zone. Beach marks, which are macroscopic markers of fatigue crack growth under variable-amplitude loading, exhibit morphologies that are closely related to the loading history. This correlation has been extensively reported for fatigue fractures in a wide range of metallic materials [50]. The fatigue crack propagation zone is relatively flat, where typical fatigue fracture characteristics such as beach marks can be observed; the instantaneous fracture zone is relatively rough, showing the microscopic morphological characteristics of ductile or brittle fracture.

The macroscopic fracture analysis above clearly identifies the crack initiation site and the overall failure zones. However, a micro-fractographic analysis via Scanning Electron Microscopy (SEM) would be essential to provide direct evidence for the micromechanical damage mechanisms, such as fatigue striation spacing (to correlate with crack growth rate) and micro-void coalescence (reflecting the damage accumulation process). Such analysis would offer the most direct validation for the damage evolution assumptions inherent in the Continuum Damage Mechanics (CDM) model employed in this study. The absence of SEM analysis in the current work is a recognized limitation, primarily due to the large size of the fractured specimens, which made standard SEM sample preparation and analysis impractical within the scope of this study focused on full-scale component testing. Nevertheless, the high accuracy of the CDM-based life predictions (with errors under 5%) provides strong indirect validation that the macroscopic damage evolution law effectively captures the dominant failure physics. Future work will prioritize micro-fractographic analysis on smaller-scale specimens to establish a direct correlation between the CDM model parameters and the microscopic damage features, thereby bridging the gap between macroscopic life prediction and microscopic damage mechanisms.

### 2.4. Consideration of Welding Residual Stresses

The potential impact of welding-induced residual stresses on the fatigue behavior, particularly during the crack initiation and early growth stages, is fully acknowledged [51]. Tensile residual stresses at the weld toe can significantly elevate the mean stress of the cyclic load, thereby accelerating fatigue damage accumulation. This effect is well-documented in the literature, with many studies aiming to explicitly model the residual stress field for fatigue assessment [52].

The present CDM model explicitly simulates damage evolution under the applied mechanical loads, with an initial condition assuming a stress-free state. This approach, which relies on the calibrated parameters to implicitly account for the mean stress effect of residual stresses, is consistent with methodologies adopted by other researchers when the primary focus is on total life prediction under a fixed manufacturing condition [53]. The demonstrated accuracy of the model’s life predictions, however, suggests that the constitutive parameters calibrated from the experimental S-N data inherently account for the net effect of the actual initial condition of the as-welded specimens, which includes the residual stress field. In essence, the calibrated model captures the macroscopic fatigue response of the structure manufactured under the specific welding procedure.

Consequently, while the model does not explicitly resolve the residual stress field, its predictive capability for the total fatigue life remains valid for the examined “as-welded” condition. It is important to note that this formulation assumes a fixed initial state. The model’s applicability to scenarios where the residual stress is altered (e.g., via post-weld treatments) would require re-calibration or explicit incorporation of the residual stress field, a challenge that has also been identified in related studies [54], which represents a key direction for future research.

## 3. Construction of a Multi-Axial Fatigue Damage Model Based on CDM

### 3.1. Basic Theory of Continuum Damage Mechanics

Continuum Damage Mechanics (CDM) is a branch of mechanics that studies the evolution of internal damage (such as the initiation and propagation of microcracks and voids) within materials under external loads. It provides the core theoretical framework for constructing multiaxial fatigue damage models.

From a thermodynamic perspective, damage can be regarded as an internal variable used to describe the degradation of a material’s mechanical properties due to the accumulation of defects. Under the continuum assumption, the impact of defects on the mechanical properties of a material is analyzed using macroscopic damage variables within a representative volume element. To quantify the degree of damage in the weld heat-affected zone (HAZ) of large welded flange shafts caused by cyclic loading, a damage variable *D* is introduced to describe the deterioration of macroscopic load-bearing capacity due to the accumulation of microscopic defects (such as microcracks and micropores). This variable is defined as the ratio of the material’s initial load-bearing area to its effective load-bearing area after damage, and is expressed as [55]

(5) $$D=1-\frac{\delta S}{\delta S_{0}}$$

where $\delta S_{0}$ is the initial load-bearing area of the material, and δS is the effective load-bearing area after the material has been damaged. When *D* is 0, the material is undamaged; when *D* tends to 1, the material is approaching complete failure. Since large welded flange shafts inevitably have initial defects such as pores and lack of fusion during the welding process, initial damage *D*_0_ is introduced. Therefore, the formula can be written as

(6) $$D=D_{0}+\frac{\delta S_{0}-\delta S_{post-D_{0}}-\delta S}{\delta S_{0}}$$

(7) $$D_{0}=\frac{\delta S_{post-D_{0}}}{\delta S_{0}}$$

where $\delta S_{post-D_{0}}$ is the effective load-bearing area occupied by the initial damage, assuming that the initial damage *D*_0_ = 0. After introducing the damage variable, based on the strain equivalence assumption, the effective stress $\tilde{\sigma }$ acting on the effective load-bearing area of the material after considering damage can be expressed as [56]

(8) $$\tilde{\sigma }=\frac{\sigma }{1-D}=\frac{\sigma }{1-\left(D_{0}+D_{evol}\right)}$$

where σ represents the nominal stress borne by the material, and $D_{evol}$ represents the damage evolution under cyclic loading. In the following sections, stresses are expressed in MPa unless otherwise stated. The total damage includes both the initial damage and the evolved damage.

### 3.2. Elastoplastic Constitutive Model Considering Damage

Considering the elastoplastic deformation characteristics of large welded flange shafts under multiaxial cyclic loading, the total strain tensor $\varepsilon_{ij}$ of the material is decomposed into two parts: the elastic strain tensor $\varepsilon_{ij}^{e}$ and the plastic strain tensor $\varepsilon_{ij}^{p}$, namely [57,58]

(9) $$\varepsilon_{ij}=\varepsilon_{ij}^{e}+\varepsilon_{ij}^{p}$$

where elastic strain is recoverable, while plastic strain is irreversible and is directly related to permanent deformation and the accumulation of damage in the material.

For the elastic constitutive relationship considering damage, by combining the generalized Hooke’s law with damage modification, we obtain [55]

(10) $$\varepsilon_{ij}^{e}=\frac{1+\upsilon }{E}\frac{\sigma_{ij}}{1-D}-\frac{\upsilon }{E}\frac{\sigma_{kk}\delta_{ij}}{1-D}$$

where υ is the Poisson’s ratio, E is the elastic modulus, $\sigma_{ij}$ is the stress tensor, $\sigma_{kk}$ is the first invariant of the stress tensor, and $\delta_{ij}$ is the Kronecker delta (when i=j, $\delta_{ij}=1$; otherwise $\delta_{ij}=0$). Since the fatigue test adopts a tension–tension (stress ratio R = 0) loading method, there is a specific tensile-compressive zone during cyclic loading. Therefore, this paper mainly uses the isotropic hardening elastoplastic constitutive equation to describe the fatigue damage process of the specimen. Regarding the isotropic hardening behavior of welded joints under cyclic loading, the following yield function is used to determine whether the material has entered plastic deformation:

(11) $$F={\left(\frac{\sigma_{ij}}{1-D}\right)}_{eq}-Q$$

where ${\left(\cdot \right)}_{eq}$ is the von Mises equivalent stress, defined as $\sigma_{eq}=\frac{1}{\sqrt{2}}\sqrt{{\left(\sigma_{1}-\sigma_{2}\right)}^{2}+{\left(\sigma_{2}-\sigma_{3}\right)}^{2}+{\left(\sigma_{3}-\sigma_{1}\right)}^{2}}$, converting a multiaxial stress state into an equivalent uniaxial stress. Here, $\sigma_{1},\sigma_{2},\sigma_{3}$ are the three principal stresses of the material under multiaxial stress (principal stresses refer to stress components that only contain normal stress (no shear stress) in a specific direction), respectively. Q represents the isotropic hardening yield surface, which is related to the accumulation of plastic strain and reflects the influence of the material’s plastic deformation history on its subsequent yield behavior. When F≥0, the material enters the plastic state; when F<0, the material remains in the elastic state.

The plastic strain rate is

(12) $${\dot{\varepsilon }}_{ij}^{p}=\frac{3}{2}\frac{\dot{\lambda }}{1-D}\frac{\left(\frac{\sigma_{ij}}{1-D}\right)s_{ij}}{{\left(\frac{\sigma_{ij}}{1-D}\right)}_{eq}}$$

where $\dot{\lambda }$ is the plastic multiplier, $s_{ij}$ is the deviatoric stress tensor. The greater the damage, the more likely plastic deformation will occur. In addition, the equivalent plastic strain increment is

(13) $$d\varepsilon_{p}=\sqrt{\frac{2}{3}d\varepsilon_{ij}^{p}:d\varepsilon_{ij}^{p}}=\frac{\dot{\lambda }}{1-D}$$

### 3.3. Fatigue Damage Evolution Model

The fatigue damage of large welded flange shafts consists of both elastic damage and plastic damage. The total damage evolution rate $\dot{D}$ can be decomposed into the sum of the elastic damage evolution rate ${\dot{D}}_{e}$ and the plastic damage evolution rate ${\dot{D}}_{p}$, that is [59]

(14) $$\dot{D}=\frac{dD}{dN}=\frac{dD_{e}}{dN}+\frac{dD_{p}}{dN}$$

For fatigue damage, considering the flange shaft is subjected to multi-axial bending–torsion loads, its evolution pattern is closely related to the multi-axial stress state, mean stress, and current damage. The evolution equation can be expressed as follows [29,31]:

(15) $${\dot{D}}_{e}=\frac{dD_{e}}{dN}=a_{D}\cdot {\left[\frac{A_{\mathrm{II}}}{\left(1-3b_{D}\sigma_{H,\mathrm{mean}}\right)\left(1-D\right)}\right]}^{\beta_{D}}$$

where N is the number of cycles, $a_{D},b_{D},\beta_{D}$ is a material parameter obtained from fitting fatigue test data; $\sigma_{H,\mathrm{mean}}$ is the mean hydrostatic stress; $A_{\mathrm{II}}$ is the amplitude of octahedral shear stress during cyclic loading, which is used as the equivalent shear stress amplitude summarizing the multiaxial stress components, and serves to quantify the magnitude of the shear stress amplitude under multiaxial cyclic loading. The expression is [60,61,62]

(16) $$A_{\mathrm{II}}=\frac{\sqrt{\frac{3}{2}\left[\left(s_{ij,\max}-s_{ij,\min}\right)\cdot \left(s_{ij,\max}-s_{ij,\min}\right)\right]}}{2}$$

(17) $$\sigma_{H,\mathrm{mean}}=\frac{\left[\max\left(\mathrm{tr}\left(\sigma \right)\right)+\min\left(\mathrm{tr}\left(\sigma \right)\right)\right]}{6}$$

where $\mathrm{tr}\left(\sigma \right)=\sigma_{11}+\sigma_{22}+\sigma_{33}$. $s_{ij,\max},s_{ij,\min}$ are the maximum and minimum deviatoric stress components, respectively, during the cyclic loading process. Based on the uniaxial high-cycle fatigue damage model, Equation (15) can be simplified as follows:

(18) $${\dot{D}}_{e}=\frac{dD_{e}}{dN}=a_{D}\cdot {\left[\frac{\sigma_{a}}{\left(1-3b_{D}\sigma_{m}\right)\left(1-D\right)}\right]}^{\beta_{D}}$$

where $\sigma_{a}$ is the stress amplitude, and $\sigma_{m}$ is the mean stress. For uniaxial bending fatigue loading with a stress ratio of R = 0, $\sigma_{a}=\sigma_{m}=\sigma_{\max}/2$.

For the plastic damage of welded flange shafts, based on Lemaître’s theory, its evolution law is directly related to the accumulated plastic strain, and the evolution equation is [56]

(19) $${\dot{D}}_{p}=\frac{dD_{p}}{dN}={\left[\frac{{\left(\sigma_{\max}^{*}\right)}^{2}}{2ES{\left(1-D\right)}^{2}}\right]}^{m}\Delta \varepsilon_{p}$$

where $\sigma_{\max}^{*}$ is the maximum equivalent stress, S is the damage energy intensity factor, and m is the damage index related to the material.

## 4. Multiaxial Fatigue Finite Element Model of Welded Specimens

### 4.1. Parameter Fitting

#### 4.1.1. Elastoplastic Constitutive Relation Parameters

In the coupled elastic damage constitutive equation and the elastic damage evolution equation, the Young’s modulus of the specimen material is 200 GPa, and the Poisson’s ratio is 0.3. The fatigue limit of large welded flange shafts under a specified stress ratio was calculated using the Basquin equation extrapolation method. The logarithmic form of the Basquin equation is [63]

(20) lgS=AlgN+B

where S is the stress range of the fatigue load, $S=\sigma_{\max}-\sigma_{\min}$; A and B are material constants. The data from groups G1–G5 were each subjected to linear fitting on logarithmic coordinates, resulting in the fatigue material constants for the bending and uniaxial fatigue of 45Mn steel welded flange shafts being A = −0.35 and B = 4.21. When the fatigue limit is set at 2 million cycles, the bending fatigue limit $\sigma_{10}$ is 101.1 MPa.

**Figure 6:**
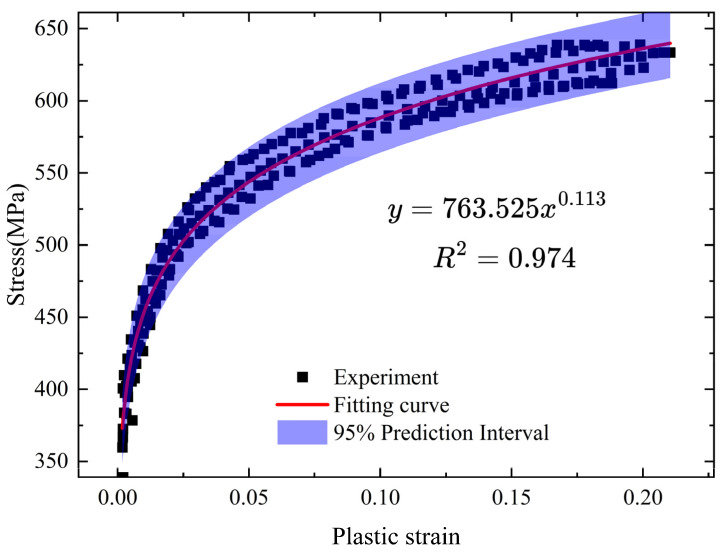
Relationship between plastic stress and strain.

The relationship between plastic strain and stress is shown in Figure 6. The data points used for establishing this relationship were obtained from the stable hysteresis loops of five replicate uniaxial tests, which were combined to form a comprehensive dataset for calibration. By performing a nonlinear least-squares regression on this combined dataset, it was found that the nonlinear strain hardening model is suitable for describing the yield flow and hardening stages of the 45Mn steel welded flange shaft. The regression yielded a high coefficient of determination (R^2^) of 0.974, indicating an excellent goodness of fit. The expression for the calibrated relationship between plastic strain and stress in uniaxial form is

(21) $$\sigma =763.525\varepsilon_{p}^{0.113}$$

where σ is the plastic stress and $\varepsilon_{p}$ is the plastic strain. As the plastic strain increases, the material stress exhibits nonlinear growth, and the rate of growth gradually slows down. The 95% prediction interval for the fitted curve is also presented in Figure 6, providing a statistical measure of the model’s prediction uncertainty for new observations. The high R^2^ value and the narrow prediction interval demonstrate the robustness of the calibrated model parameters.

For the case of multiaxial stress, the yield strength *Q* represents the size of the yield surface and is a function of the instantaneous equivalent plastic strain [64]. Then, based on the isotropic hardening model, the yield surface *Q* of 45Mn steel can be expressed as

(22) $$Q=763.525{\left({\bar{\varepsilon }}_{\mathrm{ij}}^{p}\right)}^{0.113}$$

#### 4.1.2. Parameters of the Fatigue Damage Evolution Model

According to Equation (15), there are three material parameters that need to be identified in the elastic damage model, namely $a_{D},b_{D},\beta_{D}$. The integral form of Equation (15) is as follows:

(23) $$N_{f}=\frac{1-{\left(1-D_{c}\right)}^{\left(1+\beta_{D}\right)}}{a_{D}\left(1+\beta_{D}\right)}{\left(\frac{A_{II}}{1-3b_{D}\sigma_{H,mean}}\right)}^{-\beta_{D}}$$

where $N_{f}$ denotes the fatigue life (the number of load cycles until the specimen fails); $D_{c}$ is the threshold value at which the unit fails, with its value range being $0\leq D_{c}\leq 1$. Generally, when $D_{c}=1$, the unit is considered to have failed, so Equation (23) can be transformed as follows:

(24) $$N_{f}=\frac{1}{a_{D}\left(1+\beta_{D}\right)}{\left(\frac{A_{II}}{1-3b_{D}\sigma_{H,mean}}\right)}^{-\beta_{D}}$$

Experimental data obtained from eight different working conditions (G1–G8) in fatigue tests, including stress amplitude ($\sigma_{a}$), mean stress ($\sigma_{m}$), and corresponding fatigue life ($N_{f}$), were used for parameter fitting of the fatigue life Equation (24). The fitting was formulated as an optimization problem with the objective of minimizing the sum of squared errors between the experimental and predicted fatigue lives. The key parameters to be identified were the damage parameter $a_{D},b_{D},\beta_{D}$, with physically meaningful bounds applied to constrain the solution ($a_{D}\in \left[10^{-15},10^{-10}\right],b_{D}\in \left[10^{-6},10^{-3}\right],\beta_{D}\in \left[2.0,5.0\right]$). The minimization was executed using the fminsearch algorithm implemented in MATLAB R2023b, with the iteration process displaying convergence status. Convergence was achieved when the change in the objective function (sum of squared errors) fell below a tolerance of 10^−6^. The parameter values in the elastic fatigue damage evolution model for the large welded flange shaft made of 45Mn steel are shown in Table 6.

In addition, in damage evolution models based on plastic strain, there are two parameters that need to be calibrated—S and m in Equation (19). By integrating Equation (19), the explicit form of the fatigue life expression can be obtained as follows:

(25) $$N_{f}=\frac{1}{2\left(2m+1\right)\Delta \varepsilon_{p}}{\left(\frac{2ES}{\sigma_{\max}^{2}}\right)}^{m}$$

Among them, the stress–strain relationship curve in the plastic stage can be obtained

(26) $$\sigma_{\max}=763.525{\left(\frac{\Delta \varepsilon_{p}}{2}\right)}^{0.113}$$

Due to the lack of experimental data support, the fitting of the parameters for the damage-considered plasticity constitutive model in this paper is obtained using numerical inversion and optimization algorithms. The parameters are repeatedly adjusted through a combination of finite element simulations and optimization algorithms until the simulation results match the engineering failure phenomena. The final optimized parameters are shown in Table 7 below. Their general applicability is further verified under different working conditions by varying the load amplitude and loading methods.

### 4.2. Calculation Methodology

**Figure 7:**
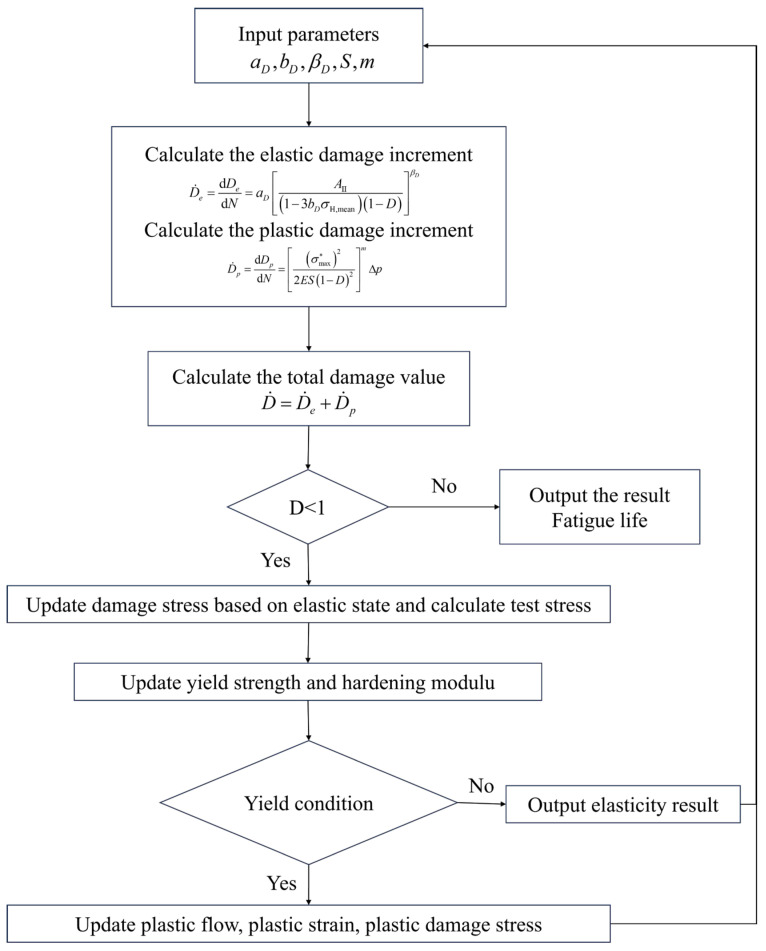
Numerical flowchart of computational methods.

This study used a self-developed user subroutine UMAT in ABAQUS for numerical simulation, and established a finite element model of a large welded flange shaft specimen based on actual fatigue tests. The flowchart of this algorithm is shown in Figure 7, and the specific steps are as follows:

(1) Initialize material parameters. Assume the initial damage is $D_{0}=0$ in the heat-affected zone of the welded flange shaft. It is important to clarify that this choice represents a computational initial condition rather than a denial of the intrinsic micro-defects in the material or the welding process. Physically, the pre-existing micro-voids, inclusions, and welding imperfections are undoubtedly present and act as initial damage. In the context of this continuum-scale model, the collective effect of these micro-defects is implicitly accounted for by the calibrated material parameters in the damage evolution law, which are fitted to the experimental S-N data obtained from the actual, imperfect welded joints. Setting $D_{0}=0$ is a conventional and necessary simplification that allows for a clear definition of the damage accumulation starting point for macroscopic life prediction. The model’s accuracy in predicting fatigue life demonstrates that this approach effectively captures the global damage evolution process initiated from these micro-defects.

(2) Call the ABAQUS solver to solve the elastic damage constitutive Equation (10) considering multiaxial stress, and calculate the stress history at each integration point under bending and bending–torsion cyclic loads. The stress tensor includes components of bending normal stress and torsional shear stress.

(3) Solve Equation (15) to calculate the increment and accumulation of elastic damage. However, since fatigue life values are large, calculating all cycles one by one would be very time-consuming. Therefore, based on previous studies, this paper adopts a jump algorithm, which assumes that the damage value and the stress–strain field remain unchanged during ΔN cycles. To ensure computational accuracy and save computational cost, ΔN is set to 1% of the fatigue life. Calculate the elastic damage evolution rate $\frac{dD_{e}}{dN}$, update the accumulated value of elastic damage $D_{e\left(i+1\right)}=D_{e\left(i\right)}+{\left(dD_{e}/dN\right)}_{\left(i\right)}\cdot \Delta_{N}$, and update the total number of cycles $N_{\left(i+1\right)}=N_{\left(i\right)}+\Delta N$.

(4) Solve Equation (18) to calculate the increment and cumulative value of plastic damage. Calculate the plastic evolution damage rate $\frac{dD_{p}}{dN}$, and update the cumulative value of plastic damage $D_{p\left(i+1\right)}=D_{p\left(i\right)}+{\left(dD_{p}/dN\right)}_{\left(i\right)}\cdot \Delta_{N}$.

(5) Add the increment of elastic damage and the increment of plastic damage to obtain the total damage increment and its cumulative value $D_{\left(i+1\right)}=D_{\left(i\right)}+{\left(dD/dN\right)}_{\left(i\right)}\cdot \Delta N$, and determine whether the damage value at any integer point has reached 1. If so, end the program and output the fatigue life $N_{\left(i+1\right)}$; otherwise, proceed to step (6).

(6) Solve the equation in step (6), update the stress according to the elastic state, and calculate the trial stress based on the von Mises yield criterion. Using the equivalent plastic strain from the previous iteration, calculate the updated yield strength.

(7) Evaluate whether the yield criterion is met. If the yield condition is not satisfied, output the elastic results from step (6) as the result and carry them over to the next cycle. If the yield condition is satisfied, proceed to step (8).

(8) Update the plastic flow, solve for the equivalent plastic strain using the Newton-Raphson method, and update the damage stress. This loop ends here, and the results are returned to the next cycle.

### 4.3. Finite Element Model

**Figure 8:**
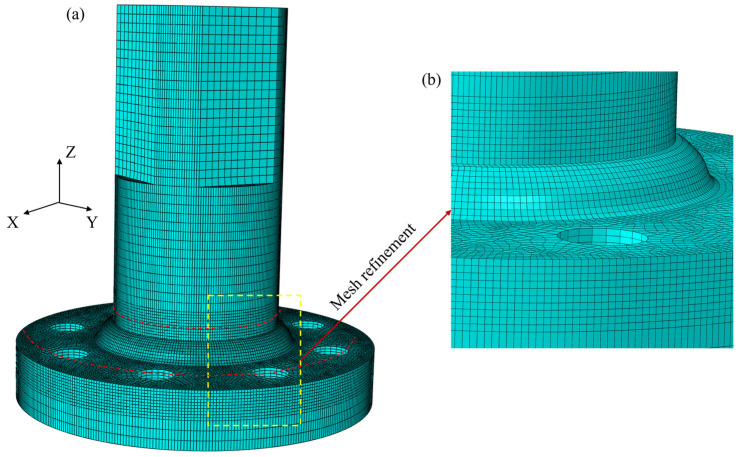
Finite Element Model Mesh: (**a**) Overall mesh; (**b**) Local mesh.

In geometric modeling, the first step is to use professional software to create a three-dimensional model of the large welded flange shaft. The finite element model is built to be completely consistent with the specimen used in the experiment. To assess mesh sensitivity, a series of meshing schemes with different element sizes were implemented before the fatigue finite element simulation. As shown in Table 8, the mesh size in the refined region (such as the weld zone and the heat-affected zone within 10 mm nearby) ranges from 1 mm to 2 mm, while the mesh size in non-refined regions (such as areas far from the weld) is between 2 mm and 4 mm. By comparing the maximum equivalent stress and the maximum bending normal stress of the structure under different mesh sizes, it was found that when the mesh size in the refined region is 1 mm and the mesh size in the non-refined region is 3 mm, further refinement of the mesh has almost no effect on the accuracy of the simulation results, but significantly increases the number of elements and reduces computational efficiency. Therefore, the mesh in the weld area and its adjacent heat-affected zone was refined to a mesh size of 1 mm, while the unrefined region used a mesh size of 3 mm. This approach ensures the accuracy of stress field simulation while greatly reducing simulation time and improving computational efficiency. The overall mesh of the specimen and the local mesh around the weld are shown in Figure 8. The element type used is the fully integrated C3D8 element. If a reduced integration element is used, it is prone to non-convergence, and the calculation accuracy of the hexahedral mesh is significantly higher than that of the tetrahedral mesh.

## 5. Result and Discussion

### 5.1. Fatigue Life Prediction

After submitting the finite element model to the ABAQUS solver and running the UMAT fatigue damage subroutine, the predicted fatigue life of the large welded flange shaft can be obtained. The fatigue life predicted by the simulation method is compared with the experimental results, as shown in Table 9. The fatigue life of the large welded flange shaft under multiple loading conditions, as predicted by the CDM method, has a maximum error within 10%. This level of accuracy is notably higher than the typical scatter observed in traditional stress-based fatigue assessment methods for welded joints, which often rely on S-N curves and can have scatter factors exceeding 3–4 [65]. To quantitatively evaluate this agreement, key statistical metrics were calculated, yielding a mean relative error (MRE) of 5.22% and a root mean square error (RMSE) of 34,210 cycles across all conditions. The scatter factor, a crucial indicator of prediction consistency in fatigue analysis, was found to be 2.18. A scatter factor below 2.5 is generally considered excellent for fatigue life prediction of welded structures, indicating high consistency of the present model [66]. This indicates that the simulation results agree well with the experimental data, and the fatigue prediction model is feasible to a certain extent [30]. The high accuracy, particularly under multiaxial loading, can be attributed to the ability of the CDM model to directly account for the nonlinear damage accumulation and the synergistic effects of bending and torsion, which are challenges for many traditional methods [67]. For pure bending conditions, the relative error between the predicted life and the experimental results is within 5%. Under combined bending and torsion conditions, although the predicted life is shortened due to the adverse effects of multiaxial loading, the relative error is still controlled within 10%. All results meet the engineering accuracy requirements for fatigue life prediction, demonstrating that the adopted CDM model can accurately capture the fatigue failure patterns under different working conditions.

**Figure 9:**
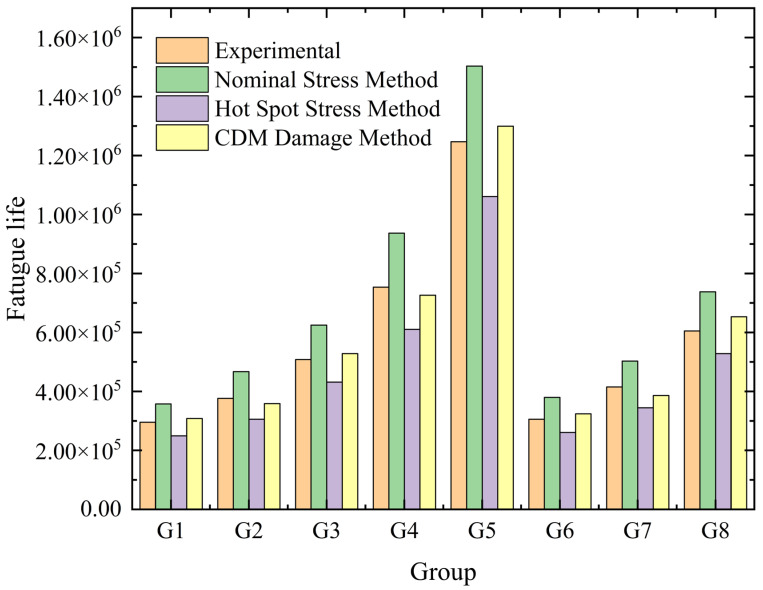
Comparison of predicted lifespan by different methods and tested lifespan.

Figure 9 compares the fatigue life predictions of the present CDM model against the conventional nominal stress and hot-spot stress methods under various loading conditions. The results clearly demonstrate the superior accuracy of the CDM approach, with errors controlled within 10%, significantly outperforming the traditional methods which exhibit larger errors and non-conservative or overly conservative tendencies.

However, to provide a more profound evaluation of the proposed model’s value, it is insightful to contextualize its performance by comparing its fundamental approach and practical implementation against other damage-based methods documented in the literature, such as Lemaitre’s formulations and the Smith–Watson–Topper (SWT) parameter. While classical continuum damage mechanics (CDM) models, like Lemaitre’s, provide a robust theoretical framework [68,69], they are often calibrated for specific loading conditions (e.g., pure bending) and may lack robustness when directly applied to the complex non-proportional multiaxial loading encountered in this study. Similarly, critical plane approaches like the SWT parameter are effective for accounting for mean stress effects [70,71], but they require precise determination of the critical plane, which is highly challenging for large-scale welded structures with geometric and microstructural discontinuities.

The model presented in this study offers a distinct practical advantage for engineering applications. It incorporates a relatively simple yet physically sound damage evolution law that was efficiently calibrated against macroscopic experimental S-N data from the actual components. This calibration strategy, implemented via a UMAT subroutine, allows the model to implicitly capture the complex combined effects of multiaxial loading, welding residual stresses, and microstructural heterogeneity, without requiring explicit—and often impractical—measurements of local strain energy or critical plane orientation on the large-scale structure. Therefore, the high accuracy shown in Figure 9, combined with its practical calibration strategy, positions the present model as a highly effective and pragmatic tool for the fatigue life assessment of large welded engineering components.

### 5.2. Analysis of Stress–Strain Field Evolution

**Figure 10:**
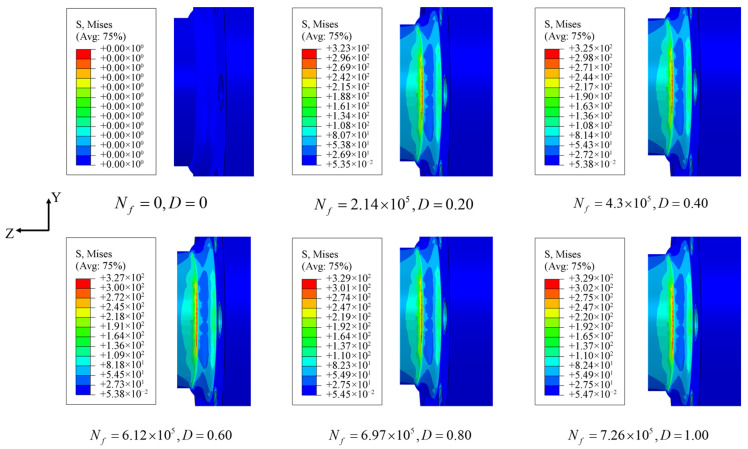
Equivalent stress distribution diagrams of flange shaft specimens under different loading cycles (taking G4 as an example).

There are significant differences compared to static analysis. Under the continuous evolution of material damage, the stress–strain field of a component subjected to cyclic external loading is in a state of constant change. As shown in Figure 10, taking the G4 specimen of a welded flange shaft under uniaxial bending fatigue as an example, the equivalent stress distribution contour maps of the structure at different cycles are presented. The results show that the stress concentration at the weld defect and its surrounding area is very pronounced. However, as the number of cycles increases, the area of stress concentration does not shift with the intensification of damage, which is a rather peculiar phenomenon. This observed “stationary” or “non-migrating” nature of the stress concentration peak is a critical finding. It contrasts with some studies on homogeneous materials or small-scale specimens, where damage evolution can lead to a redistribution of stress, causing the peak stress to migrate to adjacent, less damaged areas [72,73]. However, our observation aligns with and provides a mechanistic explanation for phenomena reported in studies of large welded structures. The fixed stress concentration peak underscores that for large-scale welded joints with inherent “dual inherent weakness,” the global structural constraint and the severity of the local stress concentrator dominate the damage process, effectively “pinning” the stress concentration at the initial notch (weld toe) throughout the fatigue life [74,75]. Generally, as material fatigue damage continues to accumulate and deteriorate in a structure, the stress value at the weld defect will gradually decrease. This phenomenon can be used to indicate the formation of macroscopic fatigue cracks. However, for large welded flange shafts, the peak stress concentration always remains fixed in the weld toe area at the joint between the weld and the flange shaft, without migrating as the number of cycles increases. The core reason lies in the “dual inherent weakness” of this region caused by both geometric discontinuity and welding-induced material heterogeneity, making it an irreplaceable “preferential stress-gathering zone” during the load transfer process.

From a geometrical perspective, there is a pronounced sharp transition feature in the weld area. According to static analysis, the geometric stress concentration factor in this region is extremely high. Such an abrupt geometric discontinuity causes the load-induced stress to be “forcibly channeled” along the load path of the flange-shaft connection towards the weld area. Even if local stiffness decreases due to fatigue damage in this area, the “stress-bearing priority” dictated by its geometry still remains significantly higher than in other regions. As a result, the external load will still be primarily borne by the weld toe area, which is more geometrically constrained, making it impossible for the stress to effectively diffuse into surrounding areas. From the perspective of welded structure characteristics, the weld toe area happens to lie at the junction between the coarse-grained zone of the heat-affected zone (HAZ) and the weld metal. During the welding thermal cycle, this area experiences high-temperature heating above 1500 °C followed by rapid cooling, resulting in grain coarsening and reduced yield strength. This “localized deterioration” in material properties, compounded with the geometric discontinuity, forms a significant “weakness superposition effect”. Although the intense thermal cycle could also involve complex dislocation activities such as dynamic recovery, the predominant microstructural change governing the fatigue behavior in this region is the coarse prior-austenite grain structure. Ultimately, the geometric abruptness and material property degradation at the weld toe together constitute an “irreversible inherent weak point,” causing this region to consistently serve as a “stable carrier” for stress concentration throughout the entire cyclic loading process and to exhibit a fixed stress concentration peak. This phenomenon also indicates that, in the structural optimization and design of large welded flange shafts, priority should be given to improving the weld root transition fillet radius and controlling welding heat input to refine the HAZ grain size, thereby weakening the locking effect of inherent structural weaknesses on the evolution of stress concentrations.

### 5.3. Fatigue Damage Evolution Analysis

**Figure 11:**
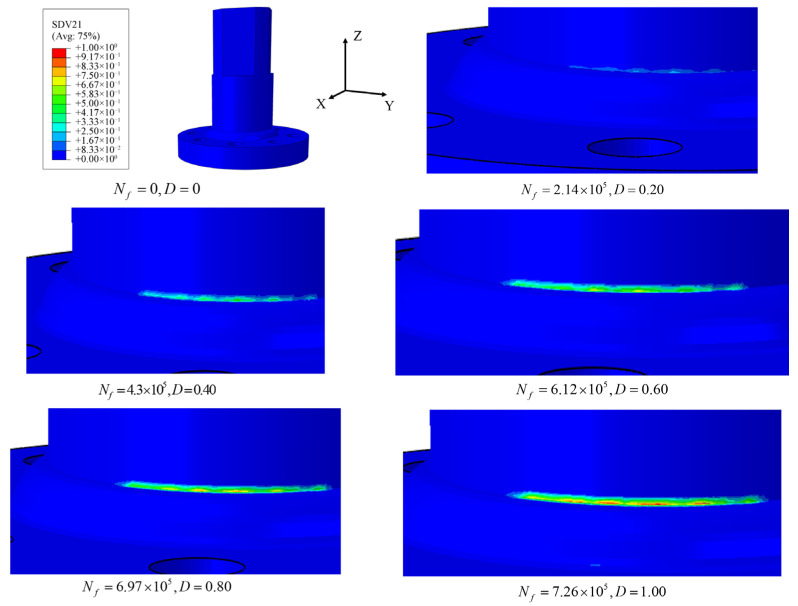
Distribution chart of weld defect edge damage values with the number of cycles (taking G4 as an example).

The significant advantage of analyzing fatigue damage problems based on the CDM model lies in its ability to accurately capture nonlinear damage evolution information. Figure 11 shows the distribution of damage values at the edge of the weld defect as the number of cycles increases. As shown in Figure 11, with an increasing number of cycles, the damage at the edge of the welding defect continuously increases until the damage reaches 1. Since stress concentration occurs at the edge of the welding defect during cyclic loading, damage tends to occur first at this location, while areas farther from the defect experience almost no damage. This precise visualization of damage localization and progression under cyclic loading is a key strength of the CDM approach, moving beyond the capabilities of traditional stress-based methods [76]. Under pure bending conditions, the form of loading is relatively singular and the stress state is closer to uniaxial, whereas under bending–torsion conditions, the combined effect of multiaxial loads leads to a more complex stress state. This results in slower damage propagation in pure bending conditions, which, compared to bending–torsion conditions, affords a longer fatigue life. This observed significant life reduction under multiaxial loading is a well-documented phenomenon. However, the present CDM model provides a more fundamental explanation by directly simulating the accelerated damage accumulation rate under the complex stress state, which is often only empirically accounted for in traditional methods using factors like the equivalent stress approach [77]. The ability of the model to replicate this key behavior without ad hoc factors strongly validates its physical basis and predictive capability for complex loading scenarios.

Macro-fractographic analysis of the failed specimens consistently revealed that fatigue cracks initiated at the weld toe on the flange shaft side. More precisely, the initiation site was located within the heat-affected zone (HAZ), immediately adjacent to the fusion line between the weld metal and the base metal. This region is characterized by a combination of geometric stress concentration (due to the weld toe) and microstructural heterogeneity (resulting from the welding thermal cycle), making it the most critical location for crack initiation. The crack initiation location was highly consistent across all tested specimens under both pure bending and combined bending–torsion loading. This reproducibility confirms that the weld toe/HAZ is the inherent structural “hot spot” governing the fatigue performance of these large welded flange shafts. The fact that the CDM model successfully predicted failure originating from this same critical location, provides strong validation for the model’s capability to capture the dominant failure mechanism, as shown in Figure 5 and Figure 11.

**Figure 12:**
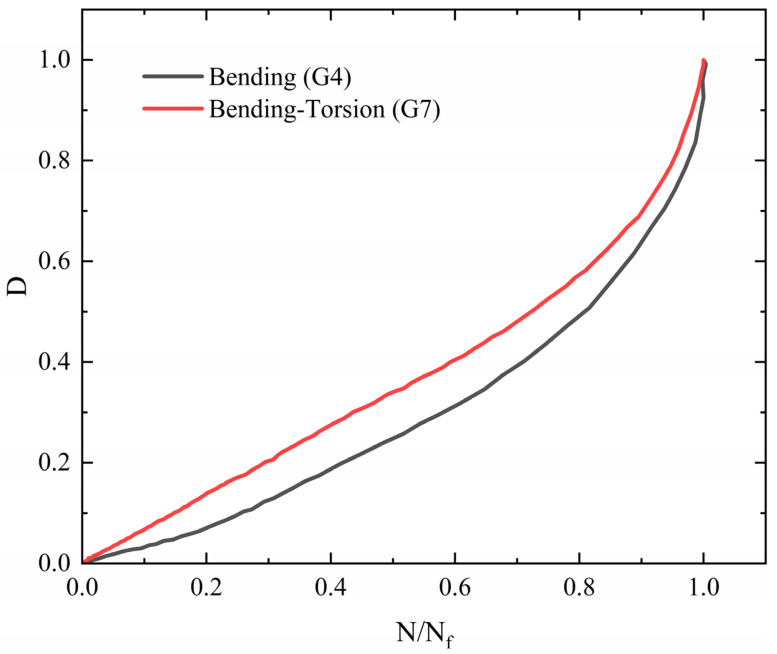
Fatigue damage accumulation curve.

Figure 12 illustrates the cumulative fatigue damage process for two typical specimens (G4 and G7). As the number of cycles increases, the early damage values remain relatively small and the growth of fatigue damage in the early stage is slow. However, as failure approaches, the damage value increases sharply. A critical observation from the simulation data is that this acceleration enters an unstable phase when the damage variable D approaches a value of 0.9. This threshold, D = 0.9, is therefore proposed as an engineering failure warning indicator. The rationale for this selection is twofold: firstly, it provides a conservative safety margin prior to final fracture (D = 1.0), allowing time for inspection or intervention; secondly, it corresponds to the clear onset of macroscopic crack growth instability in the model, signaling a rapid decline in residual life. In the pure bending tensile stage, due to the presence of geometric or mechanical discontinuities at the critical point, a stress concentration effect arises, making the stress level at this location the highest and causing damage to accumulate here first and to the greatest extent. Since damage evolution is closely related to the stress state, damage mainly concentrates at this critical location. As the number of cycles increases, the damage value at this location gradually accumulates and the growth rate accelerates; the slope of the relationship curve becomes steeper, indicating that the damage D evolves at an accelerating rate with increasing cycles, and the load-bearing capacity of the component correspondingly declines more rapidly.

In comparison, under bending–torsion conditions, the coupling of multiaxial loads makes the effect of stress concentration and the superposition of multiaxial stresses at the critical point more pronounced, leading to a faster initial rate of damage accumulation and a more evident subsequent trend of accelerated evolution. Stiffness also degrades more rapidly, further reflecting the promoting effect of multiaxial loads on damage evolution. The fatigue damage evolution law presented by CDM is highly nonlinear. Its fundamental characteristic is the shorter the fatigue life, the faster the rate of damage accumulation.

### 5.4. Analysis of the Influence of Plastic Damage Under High-Cycle Fatigue on Life Prediction Results

**Figure 13:**
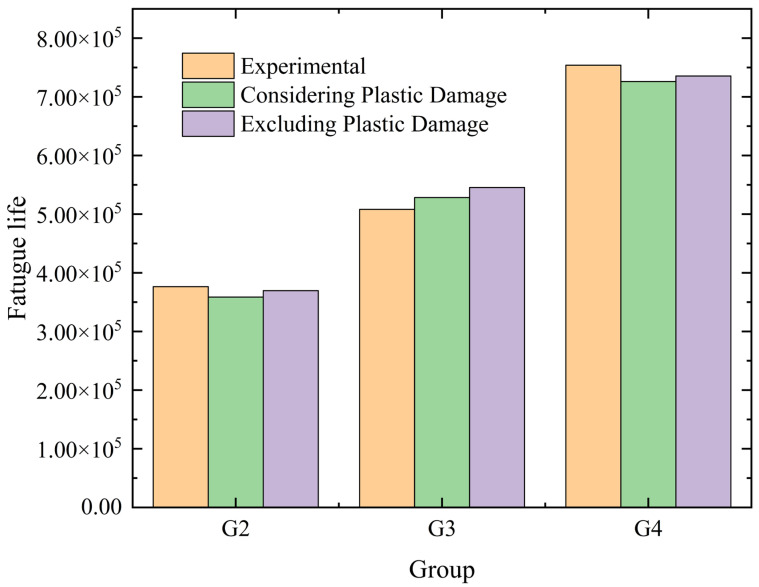
Fatigue life prediction of the CDM model considering and excluding plastic damage.

Given that the fatigue test results in this study are mostly within the high-cycle fatigue range, in order to clarify whether plastic damage evolution needs to be considered in life prediction models based on Continuum Damage Mechanics (CDM), two models were established: “the full CDM model considering plastic damage” and “the simplified CDM model excluding plastic damage.” Based on CDM theory, the full model simultaneously couples the evolution equations of elastic and plastic damage, calculating elastic damage via the accumulation of elastic strain energy and quantifying plastic damage through microplastic deformation. The simplified model retains only the elastic damage evolution equation, assuming that the accumulation of plastic damage can be ignored under high-cycle fatigue. Three groups of typical stress amplitudes were selected (G2 (180 MPa), G3 (160 MPa), G4 (140 MPa)), and life predictions were conducted using both models, with the results compared to experimentally measured lives under the corresponding stress amplitudes, as shown in Figure 13. The results indicate that at a stress amplitude of 180 MPa, the predicted life of the full model is 358,540 cycles, the simplified model 369,816 cycles, and the experimentally measured life 376,358 cycles, with prediction errors of 4.73% and 1.74%, respectively. At a stress amplitude of 160 MPa, the full model predicts a life of 528,476 cycles, the simplified model 545,330 cycles, and the experimentally measured life is 508,168 cycles, with errors of 4% and 7.31%, respectively. At a stress amplitude of 140 MPa, the full model predicts a life of 726,269 cycles, the simplified model 735,498 cycles, and the experimentally measured life is 753,967 cycles, with errors of 3.67% and 2.45%, respectively.

Under high-cycle fatigue, the error between the predicted life and experimental values for the CDM models that consider and exclude plastic damage is less than 10%. Moreover, the maximum prediction difference between the two models is only 3.31%, which has a negligible impact on the final life assessment results. At the same time, the simplified model eliminates the need for iterative calculations of plastic damage, reducing the analysis time for a single stress amplitude from 22 h in the full model to 4.5 h, significantly improving computational efficiency.

### 5.5. CDM Model Applicability Analysis and Outlook

The findings presented in Section 5.4 confirm that excluding plastic damage is a valid and efficient simplification for the high-cycle fatigue (HCF) conditions examined in this study. However, the applicability of this approach is bounded by specific physical constraints, and several limitations of the current work should be acknowledged to guide future research.

Firstly, the validity of the simplified model is confined to the HCF regime, where applied stresses are predominantly elastic and damage accumulation is governed by elastic strain energy. Consequently, the model’s exclusion of plastic damage becomes a critical limitation under low-cycle fatigue (LCF) conditions, where macroscopic plasticity dictates damage evolution. Applying the model under such conditions would yield non-conservative life predictions. Therefore, a primary direction for future work is to develop a unified fatigue damage model that incorporates both elastic and plastic damage mechanisms, calibrated through dedicated LCF experiments.

Secondly, the current CDM model is developed for the as-welded condition and does not explicitly incorporate the effects of welding residual stress. This is a recognized limitation, as tensile residual stresses can significantly shorten the actual fatigue life. The integration of an initial residual stress field, either through simulation or measurement, into the CDM framework is essential for further improving prediction accuracy.

Furthermore, the model does not account for the potential benefits of post-weld improvement techniques, such as stress relief heat treatments or mechanical surface treatments like shot peening. Extending the model to simulate the life-enhancement effects of these technologies by modifying the initial state variables (e.g., residual stress) represents a valuable avenue for increasing its practical utility in optimal design.

Lastly, the parameter fitting for the plastic constitutive model, particularly under complex loading paths, requires more extensive experimental validation to ensure its robustness across a wider range of conditions.

In summary, while the present model provides an effective solution for HCF life prediction of large welded flange shafts, its future extension to encompass LCF, welding residual stresses, life-enhancement techniques, and broader validation will undoubtedly strengthen its comprehensiveness and accuracy.

## 6. Conclusions

This study focuses on large welded flange shafts as the primary test specimens, carrying out multi-condition fatigue testing and theoretical modeling, with an emphasis on high-cycle fatigue life prediction and failure mechanism analysis. By conducting two types of fatigue tests—pure bending uniaxial and bending–torsion multiaxial—combined with fracture surface analysis, the study clarifies the origin of failure and the influence patterns of different working conditions on service life. A fatigue life prediction model is constructed based on Continuum Damage Mechanics (CDM), alongside analyses of the impact of plastic damage and studies on damage evolution under high-cycle fatigue, enabling accurate prediction of flange shaft fatigue life and early failure warning under both uniaxial and multiaxial loading conditions. The main conclusions of this study are as follows:

(1) Pure bending uniaxial and bending–torsion multiaxial fatigue tests were carried out on large welded flange shafts. Results show that the weld toe notch area is the common origin of failure in both conditions; under bending–torsion multiaxial loading, fatigue life is reduced by 35%~42% compared to pure bending, and both the spacing of fatigue striations and the instant fracture area on the fracture surface are significantly larger, proving that multiaxial loading accelerates local damage evolution through stress coupling effects. This provides experimental support for research on multiaxial fatigue failure mechanisms.

(2) A CDM-based damage evolution equation reflecting the failure mechanism of welded structures was constructed and calibrated against the experimental data. The corresponding UMAT subroutine was developed and embedded into a finite element platform, enabling numerical simulation of the damage evolution process. The model achieved accurate life prediction for both uniaxial and multiaxial loading conditions, with errors under 5%, demonstrating a significant improvement over traditional methods.

(3) A systematic comparison with traditional methods (e.g., nominal and hot spot stress) was performed, clarifying the superiority of the CDM model. The model not only provides higher accuracy but also visualizes the damage accumulation process, offering a novel approach for the life assessment of welded structures.

(4) The validated model provided theoretical and technical references for engineering design. It was found that for high-cycle fatigue, a simplified model ignoring plastic damage can greatly improve computational efficiency with acceptable accuracy. Furthermore, a damage threshold (D = 0.9) was identified as an early warning indicator for fatigue failure, which is crucial for structural health monitoring.

## Figures and Tables

**Table 1:** Chemical composition of base material and filler material (wt.%) [38].

Material	C	Si	Mn	P	S	Ni	Cr	Cu	Mo	V	Fe
45Mn steel	0.46	0.24	0.71	0.016	0.006	-	0.0131	0.0097	-	-	Bal.
E5015	0.15	0.90	1.60	0.035	0.035	0.30	0.20	-	0.30	0.08	Bal.

**Table 2:** Material Parameters of 45Mn Steel Welded Flange Shaft.

Material	Elastic Modulus	Poisson’s Ratio	Yield Strength	Tensile Strength	Elongation After Fracture
45Mn steel	200 GPa	0.3	390 MPa	634 MPa	23.5%

**Table 3:** Fatigue test results of welded flange shaft specimen.

Group	Maximum Bending Normal Stress (Mpa)	Maximum Torsional Shear Stress (MPa)	Mean Stress (MPa)	Code	Fatigue Life	Mean Fatigue Life
G1	200	-	100	G1-1	315,781	295,763
				G1-2	287,549	
				G1-3	283,959	
G2	180	-	90	G2-1	397,457	376,358
				G2-2	384,569	
				G2-3	347,048	
G3	160	-	80	G3-1	521,978	508,168
				G3-2	478,695	
				G3-3	523,831	
G4	140	-	70	G4-1	735,154	753,967
				G4-2	769,541	
				G4-3	757,206	
G5	120	-	60	G5-1	1,279,657	1,246,978
				G5-2	1,203,984	
				G5-3	1,257,293	
G6	180	180	90	G6-1	315,786	305,874
				G6-2	287,451	
				G6-3	314,385	
G7	160	160	80	G7-1	445,168	415,314
				G7-2	421,397	
				G7-3	379,377	
G8	140	140	70	G8-1	624,573	605,127
				G8-2	598,412	
				G8-3	592,396	

All tests were conducted at a constant frequency of 4 Hz, and stress ratio R = 0.

**Table 4:** Statistical parameters of fatigue test results for welded flange shafts.

Stress Level (MPa)	200	180	160	140	120
$\bar{x}$	5.470	5.575	5.706	5.877	6.096
SE	0.174	0.031	0.022	0.010	0.014
$\upsilon_{x_{i}}$	0.032	0.006	0.004	0.002	0.002

**Table 5:** Numerical summary of the S-N curve fit and its uncertainty intervals at characteristic stress levels.

Group	Stress Level (MPa)	Predicted Life (Cycles)	95% Confidence Interval (Cycles)	95% Prediction Interval (Cycles)
G1	200	284,577	[267,855, 298,497]	[246,439, 324,437]
G2	180	384,535	[363,826, 393,517]	[329,427, 432,251]
G3	160	538,676	[512,532, 547,124]	[465,154, 604,624]
G4	140	788,455	[746,752, 800,505]	[678,132, 877,058]
G5	120	1,224,767	[1,128,054, 1,269,200]	[1,040,807, 1,375,593]

**Table 6:** Parameter values in the elastic fatigue damage evolution model of large welded flange shafts made of 45Mn steel.

$a_{D}$	$b_{D}$	$\beta_{D}$
2.392 × 10^−9^	0.008	1.042

**Table 7:** Parameter values in the plastic fatigue damage evolution model of large welded flange shafts made of 45Mn steel.

S	m
4.3521	3.5892

**Table 8:** Mesh sensitivity validation.

Mesh Size (mm)		Number of Units	Equivalent Stress (MPa)	Bending Normal Stress (MPa)
Non-Refined	Refined			
4	2	30,042	121.3	114.5
4	1.5	50,220	143.5	135.6
4	1	107,261	162.3	157.1
3	2	38,146	108.6	93.3
3	1.5	58,184	145.6	139.7
3	1	118,911	167.2	132.3
2	1.5	96,941	141.1	113.8
2	1	159,279	163.4	131.6

**Table 9:** Comparison of fatigue life between experiment and CDM method simulation.

Group	Experimental Data	Simulated Data	Error
G1	295,763	308,051	4.15%
G2	376,358	358,540	4.73%
G3	508,168	528,476	4.00%
G4	753,967	726,269	3.67%
G5	1,246,978	1,299,327	4.20%
G6	305,874	324,206	6.01%
G7	415,314	386,282	7.02%
G8	605,127	653,588	8.01%
Statistical Summary	-	-	Mean Relative Error (MRE): 5.22% Root Mean Square Error (RMSE): 34,210 Scatter Factor: 2.18

## Data Availability

The original contributions presented in this study are included in the article. Further inquiries can be directed to the corresponding author.

## Nomenclature

n	Sample size
CE	Carbon Equivalent
$\bar{x}$	Mean value
SE	Standard deviation
$\upsilon_{x_{i}}$	Coefficient of variation
D	Damage variable
$D_{0}$	Initial damage
δS	Effective bearing area of damaged materials
$\delta S_{0}$	Initial bearing area of materials
$\delta S_{post-D_{0}}$	Effective bearing area occupied by the initial damage of materials
$\tilde{\sigma }$	Effective stress borne by the effective bearing region of materials after considering damage
σ	Nominal stress borne by materials
$D_{evol}$	Evolved damage under cyclic loading
$\varepsilon_{ij}$	Total strain tensor
$\varepsilon_{ij}^{e}$	Elastic strain tensor
$\varepsilon_{ij}^{p}$	Plastic strain tensor
υ	Poisson’s ratio
E	Elastic modulus
$\sigma_{ij}$	Stress tensor
$\sigma_{kk}$	First invariant of the stress tensor
$\delta_{ij}$	Kronecker delta
${\left(\cdot \right)}_{eq}$	Von Mises equivalent stress
${\dot{\varepsilon }}_{ij}^{p}$	Plastic strain rate
$\dot{\lambda }$	Plastic multiplier
$s_{ij}$	Deviatoric stress tensor
$\dot{D}$	Total damage evolution rate
${\dot{D}}_{e}$	Elastic damage evolution rate
${\dot{D}}_{p}$	Plastic damage evolution rate
N	Number of cycles
$a_{D},b_{D},\beta_{D},S,m$	Material parameters
$\sigma_{H,\mathrm{mean}}$	Mean hydrostatic stress
$A_{\mathrm{II}}$	Octahedral shear stress amplitude
$s_{ij,\max},s_{ij,\min}$	Maximum or minimum deviatoric stress component during cyclic loading
$\sigma_{a}$	Stress amplitude
$\sigma_{m}$	Mean stress
